# Association of the *FTO* rs9939609 variant with glycemic control based on fasting glucose

**DOI:** 10.1186/s13104-026-07933-0

**Published:** 2026-07-07

**Authors:** Nicolas Fragoso-Bargas, Rocio Vanessa Escarcega-Castro, Irma Quintal-Ortiz, Ligia Vera-Gamboa, Guillermo Valencia-Pacheco, Nina Valadez-Gonzalez

**Affiliations:** 1 Department of Clinical Science, Mohn Center for Diabetes Precision Medicine, University of Bergen, Yucatan, 5009 Merida, Norway; 2 Hematology Laboratory, Regional Research Center “Dr. Hideyo Noguchi”, Autonomous University of Yucatán, 97225 Mérida, Yucatán Mexico

**Keywords:** T2D, Glycemic control, rs9939609, OHA, FTO, SNV

## Abstract

**Background:**

Type 2 diabetes (T2D) affects 11.1% of the global population, underscoring the need for biomarkers that help characterize glycemic control status among treated individuals. We evaluated the association between the *FTO* variant rs9939609‑A and glycemic control in a Mexican population.

**Methods:**

A total of 174 individuals living with T2D from Mérida and Sisal, Yucatán, were included, of whom 85% were receiving oral hypoglycemic agents as main treatment. Glycemic control was defined cross‑sectionally as good (≤ 130 mg/dL, *n* = 63) or poor (> 130 mg/dL, *n* = 111) with fasting glucose. Linear mixed models incorporating relevant covariates and a family random intercept were used. Effect size estimates were transformed to logit odds ratios.

**Results:**

After adjustment for age, sex, BMI, years since T2D diagnosis, and treatment, the minor allele of rs9939609 (A) was associated with an increased risk of poorer glycemic control, reaching significance under both the additive (OR = 1.145 [1.003–1.307], *p* = 0.047) and recessive (OR = 1.514 [1.026–2.234], *p* = 0.038) models. In an alternative model adjusting for waist circumference instead of BMI, the effect of rs9939609‑A in the additive model was slightly attenuated (OR = 1.135 [0.994–1.297], *p* = 0.063), while the recessive model remained significant (OR = 1.486 [1.010–2.189], *p* = 0.046).

**Conclusions:**

rs9939609-A was associated with poorer glycemic control in this exploratory cohort, but replication in larger and ancestrally characterized samples is required.

## Introduction

Type 2 diabetes (T2D) is a multifactorial disease characterized primarily by insulin resistance, followed by gradual dysfunction of pancreatic β‑cells, which together lead to chronic hyperglycemia [1]. The global prevalence of diabetes is 11.1%, and this number is projected to increase by approximately 45% by 2050 [2], i.e., to about 16.1%. This growing burden highlights the need to better understand the biological determinants of glycemic control, including genetic factors that influence glycemic control status among treated individuals and optimal glucose regulation.

Diabetes is generally managed with diet and life-style changes and, if necessary, with oral hypoglycemic agents (OHA) such as metformin and sulfonylureas [3]. Treatment is considered successful when the patient achieves adequate glycemic control [4]. According to standard clinical definitions, good glycemic control includes a fasting plasma glucose ≤ 130 mg/dL [5]. When adequate glycemic control cannot be achieved with OHA, insulin therapy, whether as monotherapy or as an adjunct to OHA, may be considered [6]. Regardless of the treatment modality, failure to meet the recommended glycemic targets is considered poor glycemic control [7].

Thus far, eight GWAS have been conducted to identify genetic factors associated with glycemic control or treatment response in T2D [8–15], but the number of associated loci remains very small compared with those identified for T2D diagnosis. While T2D GWAS have identified ~ 1,300 loci using sample sizes of approximately 2.5 million individuals [16], GWAS on glycemic control have been considerably underpowered, including only ~ 300–4,000 participants, limiting discovery. As a result, only around 10 SNVs have been robustly associated with glycemic control and related phenotypes [8, 11–15]. Consequently, the genetics underlying glycemic control in T2D remain far less explored than the genetic architecture of T2D onset itself. In the absence of well-powered GWAS, examining candidate genes within previously validated T2D loci may represent an immediate and pragmatic strategy for identifying variants associated with glycemic control.

The fat mass and obesity‑associated gene (*FTO*) spans approximately 400 kb and is located on chromosome 16q12.2 [17]. One of its most widely studied genetic variants, rs9939609 (T > A), has been consistently associated with body mass index (BMI) and obesity across diverse populations [18, 19]. However, rs9939609‑A has also shown consistent associations with T2D [20], and in Northern European [21], Vietnamese [22] and Southeastern Mexico [23] populations, this association appears to be independent of BMI. In the same Southeastern Mexican population, where Mayan ancestry is prevalent, rs9939609‑A was also associated with fasting glucose levels [23], further supporting a potential role for *FTO* in glycemic regulation. Given its well‑documented associations with T2D risk and fasting glucose, rs9939609‑A represents a strong biological candidate for contributing to glycemic control among individuals with established T2D.

Therefore, the aim of this study is to assess cross-sectional associations between rs9939609‑A and clinically defined glycemic control (fasting plasma glucose ≤ 130 mg/dl) in a well-characterized Mexican population, leveraging detailed data on diabetes treatment and clinical management.

## Materials and methods

### Study population

The study included adults with T2D from two locations in Yucatan. The first population consisted of individuals enrolled in a case-control study conducted in San Jose Tecoh, Merida, where participants were recruited during 2018 and 2019 [23]. From this study, we included 92 individuals living T2D who were diagnosed according to Mexican clinical guidelines (NOM-015-SSA2-2010), defined as fasting plasma glucose ≥ 126 mg/dL, random glucose ≥ 200 mg/dL, or ≥ 200 mg/dL two hours after a 75 g oral glucose load. Individuals were older than 20 years of any sex and born in Merida.

The second population consisted of individuals from a population-based study conducted in Sisal, Yucatan, between 2010 and 2012. Eligible individuals were born in Sisal with deep local family roots spanning at least three generations [24]. Recruitment focused on people with T2D and their metabolically healthy relatives, all older than 20 years. Exclusions included pregnancy and diabetes types other than T2D. In total, 106 individuals with T2D and 145 without T2D were enrolled, with 99 and 133, respectively, having complete genotyping and age data.

From the 92 individuals with T2D from San Jose Tecoh and the 99 from Sisal, only those with full clinical and genetic information were kept. Individuals with fasting glucose values < 70 mg/dL (*n* = 4) were excluded to maintain a more homogeneous definition of glycemic control. Hypoglycaemic values were not considered, as they represent a distinct physiological and clinical state outside the normoglycemia-hiperglycemia spectrum evaluated in this study. The final sample included 81 participants from San Jose Tecoh and 93 from Sisal, giving a total of 174 individuals. The ages of the participants ranged from 32 to 84 years in the good control group and from 25 to 87 years in the poor control group.

### Anthropometrics and biochemical clinical measurements

Age, years living with T2D, waist circumference, height, and weight were recorded at recruitment in both study populations, and body mass index (BMI) was calculated from these measurements. Fasting plasma glucose was measured in all participants using the same Roche Cobas c111 analyzer (Roche Diagnostics Ltd., Rotkreuz, Switzerland) following the manufacturer’s guidelines.

### Cross-sectional determination of glycemic control categories

Glycemic control was assessed according to the Mexican NOM 015 SSA2 2010 clinical criteria, which, among its definitions, determines good control as a fasting plasma glucose ≤ 130 mg/dL and poor control as values at or above this threshold. In Mexico, fasting glucose–based assessment of glycemic control, in addition to be officially supported by guidelines, it is also widely used due to its accessibility and cost-effectiveness. In clinical practice, it can be measured in plasma or self-monitored by patients using point-of-care devices. Based on this criterion, 111 participants were classified as having poor glycemic control and 63 as having good control.

### Genotyping

DNA was extracted from peripheral blood using the Macherey-Nagel^®^ kit (Macherey-Nagel GmbH & Co. KG, Düren, Germany) following the manufacturer’s protocol. Real‑time PCR genotyping of the rs9939609 variant was performed in all participants using the ECO™ Illumina^®^ platform. A TaqMan^®^ allele discrimination assay with the specific probe C__30090620_10 (Applied Biosystems, Foster City, CA, USA) was used, following the manufacturer’s recommended protocol. Each run included positive and negative controls.

### Statistical analyses

Hardy Weinberg equilibrium was evaluated in the full sample and in the poor and good control groups using the Exact test of Wigginton [25], which maintains proper type I error control in both large and small datasets. All computations were done with the “HardyWeinberg” R package version 1.7.9 [26–28].

Genetic association analyses were designed by considering the major T allele as the reference. The additive model was used as the primary analytical approach (TT = 0, AT = 1, AA = 2), while the dominant (TT = 0, AT/AA = 1), recessive (TT/AT = 0, AA = 1), and overdominant (TT/AA = 0, AT = 1) models were included as sensitivity analyses to explore whether the observed association is better explained by a specific underlying inheritance pattern. Individuals in San Jose Tecoh were unrelated, while the Sisal sample included several families. To account for relatedness and avoid confounding, mixed linear regression with family as a random intercept was used. Linear models in general handle sparse categories and are widely used for binary traits such as T2D [29]. Estimates were exponentiated to approximate odds scale effects.

Five models were built: a crude model, a model adjusted for age and sex, a model adjusted for age, sex, years with T2D, and treatment, a fourth model with the same adjustments plus BMI, and a fifth model replacing BMI with waist circumference. Treatment originally had seven categories: only diet control, metformin, glibenclamide, metformin with glibenclamide, pioglitazone, insulin monotherapy, and insulin with metformin. Because some categories had very small sample sizes, they were grouped into three: only diet control, OHAs, and insulin use. These three groups were used in modeling. Statistical significance was set at *p* < 0.05, and *p* < 0.1 was considered a marginal association. All analyses were performed in R Studio version 2025.09.1 plus 401.

## Results

### Population characteristics

Table 1 presents the population characteristics. Of the 174 individuals included in this study, 111 were classified in the poor control group and 63 in the good control group. The mean age of the population was 57.29 years, with individuals in the poor control group being on average 55.95 years and those in the good control group 59.65 years. The vast majority (85%) of the population were treated with OHAs alone. Approximately 68% of individuals were female. No individuals included in this study had missing values except for a single value for waist circumference from the poor control group.

Regarding the distribution of relatedness within the sample, most individuals (*n* = 127) were singletons, while the remaining participants were related, with a median of 2 individuals per family [range: 2–5]. In the good control group, 55 individuals were singletons, while in the related individuals we observed a median family size of 2 [range: 2–2]. In the poor control group, 91 individuals were singletons, while related individuals had a median family size of 2 [range: 2–4].

**Table 1:** Clinical and demographic characteristics of the studied population

Phenotype	All (*n* = 174)	Poor control (*n* = 111)	Good control (*n* = 63)
Age	57.29 (11.66)	55.95 (11.76)	59.65 (11.20)
Female sex (% of all studied population)	119 (68.39)	74 (67)	45 (71.43)
BMI (kg/m^2^)	32.05 (6.96)	32.76 (7.15)	30.80 (6.48)
Waist circumference (cm)	101.42 (12.39)	102.92 (12.36)	98.81 (12.10)
Fasting glucose (mg/dL)	168.30 (64.81)	173.37 (71.47)	108.56 (15.40)
Years with T2D	9.90 (7.62)	9.93 (7.52)	9.85 (7.87)
**Only diet control**	11 (6.32)	9 (8.11)	2 (3.18)
**Oral hypoglycemics**	148 (85.06)	90 (81.10)	58 (92.06)
Metformin	42 (24.14)	21 (18.92)	21 (33.33)
Glibenclamide	25 (14.37)	13 (11.71)	12 (19.05)
Glibenclamide and Metformin	80 (45.98)	56 (50.45)	24 (38.10)
Pioglitazone	1 (0.57)	0 (0)	1 (1.59)
**Insulin use**	15 (8.62)	12 (10.81)	3 (4.48)
Insulin monotherapy	7 (4.02)	4 (3.60)	3 (4.76)
Insulin and metformin	8 (4.60)	8 (7.21)	0 (0)

Data are mean(sd) for quantitative variables and n (%) for categorical variables. Treatment scheme supergroups are shown in bold, followed by their specific subcategories

### Frequency distribution of rs9939609 in the studied population

The frequency distribution of rs9939609 is presented in Table 2. Across the entire population, the minor allele (A) had a prevalence of 17.82%, with 20.72% in the poor control group and 12.70% in the good control group. Notably, the AA homozygous mutated genotype was absent in the good control group. No deviations from HW equilibrium were observed in the overall study population (*p* = 0.796) or within the good (*p* = 0.561) and poor (*p* = 0.580) glycemic control groups.

**Table 2:** Genotype and allele frequencies (%) of the rs9939609 variant

Genotype and Allele frequencies							
Genotype	All	Poor control	Good control	Allele	All	Poor control	Good control
TT	118 (67.82)	71 (63.97)	47 (74.60)	T	286 (82.18)	176 (79.28)	110 (87.30)
AT	50 (28.74)	34 (30.63)	16 (25.40)	A	62 (17.82)	46 (20.72)	16 (12.70)
AA	6 (3.44)	6 (5.40)	0 (0)				

### Association between rs9939609-A and glycemic control

Association analyses are presented in Table 3. In the crude (unadjusted) analysis, we observed a statistical trend (*p* < 0.10) in both the additive and recessive models, suggesting that A-allele carriers may have an increased risk of poor glycemic control. After adjustment for age and sex, both models reached the conventional significance threshold (*p* < 0.05). In the additive model, each additional copy of the A allele was associated with a 1.15-fold higher likelihood of poor glycemic control. Under the recessive model, individuals homozygous for the A allele (AA) showed a 1.49-fold increased risk of poor glycemic control compared with carriers of the T allele (AT or TT). The association became marginal in both the additive (OR = 1.38; *p* = 0.059) and recessive (OR = 1.45; *p* = 0.062) models when further adjusting for years with T2D and treatment. Nevertheless, after further adjustment for BMI, both models strengthened and became statistically significant (*p* < 0.05), with ORs of 1.15 and 1.51, respectively. However, when using waist circumference instead of BMI, the effect size of the additive model weakened, and the association became borderline significant (OR = 1.14, *p* = 0.063), while the effect size of the recessive model persisted (OR = 1.49, *p* = 0.046).

**Table 3:** Association analyses of rs9939609 with glycemic control

Association analyses with glycemic control								
Model	Additive OR (IC)	*p*	Dominant OR (IC)	*p*	Overdominant OR (IC)	*p*	Recessive OR (IC)	*p*
Unadjusted	1.132 (0.994–1.290)	0.063	1.119 (0.961–1.304)	0.151	1.061 (0.905–1.243)	0.466	1.455 (0.985–2.148)	0.061
Age + Sex	1.148 (1.007–1.308)	**0.040**	1.134 (0.972–1.322)	0.111	1.068 (0.912–1.252)	0.414	1.493 (1.014–2.198)	**0.044**
Age + Sex + Treatment + Years with diabetes	1.380 (0.996-1.300)	0.059	1.125 (0.962–1.316)	0.142	1.063 (0.904–1.250)	0.461	1.451 (0.984–2.143)	0.062
Age + Sex + Treatment + Years with diabetes + BMI	1.145 (1.003–1.307)	**0.047**	1.128 (0.965–1.318)	0.132	1.059 (0.902–1.244)	0.485	1.514 (1.026–2.234)	**0.038**
Age + Sex + Treatment + Years with diabetes + Waist	1.135 (0.994–1.297)	0.063	1.118 (0.996–1.308)	0.166	1.051 (0.894–1.236)	0.547	1.486 (1.010–2.189)	**0.046**

Associations with *p* < 0.05 are highlighted in bold. Models were defined as follows using T as the reference allele: additive (TT = 0, AT = 1, AA = 2), dominant (TT = 0, AT/AA = 1), recessive (TT/AT = 0, AA = 1), and overdominant (TT/AA = 0, AT = 1). The additive model was primary; others were used to explore the underlying inheritance pattern of the association

## Discussion

We found that the rs9939609-A allele was associated with clinically defined poor glycemic control. A previous report suggested a similar pattern using simple unadjusted analyses [30], but to our knowledge, few studies have evaluated this association while adjusting for relevant covariates, such as years living with T2D, treatment scheme, BMI and waist circumference. Overall, the additive model suggested that carriers of the A allele were more likely to exhibit poor glycemic control (up to 1.15 after BMI adjustment), with a stronger effect observed under the recessive model, where AA homozygotes showed up to a 1.51-fold increased risk. However, these results should be interpreted with caution, as no AA homozygotes were observed in the good control group, potentially inflating the effect estimate and limiting the stability of the comparison.

Adjustment for age, sex, years with T2D, and treatment yielded a marginal association between rs993960-A and glycemic control. However, adding BMI to this model strengthened the effect size and the association became significant in the main additive model, and in the recessive model. The strengthening of the signal after BMI adjustment indicates a BMI independent genetic effect, suggesting that BMI may mask part of the association. This is partly in line with our previous study, in which the A allele of rs9939609 was associated with T2D onset independently of BMI [23]. Moreover, this BMI independent association with T2D has also been reported in Vietnamese [22] and Northern European [21] populations. Although we observed an association independent of BMI, replacing BMI with waist circumference led to an attenuation of the association in the additive model, while it remained in the recessive model. This suggests that adiposity may still partly account for the association. However, given that the attenuation was modest, the results were borderline significant, and the effect remained in the recessive model, it is possible that the association could reach statistical significance with a larger sample size. Taken together, these findings support the notion, previously proposed in our work [23], that *FTO* may influence T2D risk through mechanisms beyond adiposity. The present results suggest that such adiposity independent pathways may also operate in the context of glycemic control, warranting further investigation but remaining outside the scope of this article.

Notably, our previous study suggested that rs9939609 does not directly influence fasting glucose in healthy individuals but may exert its metabolic effects through T2D‑related pathways [23]. The current finding that the A allele is associated with poorer glycemic control among individuals with T2D is consistent with this interpretation, indicating that the variant’s impact on fasting glucose manifests under conditions of impaired glucose regulation rather than in metabolically healthy states. However, these conclusions remain a potential hypothesis due to the cross-sectional nature of the associations observed previously.

Most participants (85%) received oral glucose lowering medications, which suggests that the association between the rs9939609 A allele and poorer glycemic outcomes likely reflects inadequate glycemic control despite active treatment. However, the study could not evaluate whether specific drugs, treatment combinations, or insulin use modify this association because the sample size limited stratified or interaction analyses. Larger studies with more detailed treatment data are needed to determine whether the rs9939609 A allele has pharmacogenomic effects related to specific glucose lowering therapies.

The study was carried out in Yucatan, a region in Southeastern Mexico with high Mayan ancestry and an admixed population [31, 32]. Although ancestry informative markers were not measured, previous work in the same region showed that the A allele of rs9939609 was associated with T2D in a direction consistent with most global studies [23]. In the present study, the same allele was also associated with poorer glycemic control. Taken together, these findings suggest that ancestry is unlikely to be a major confounder, though future studies including ancestry informative markers would help confirm this.

## Conclusions

In conclusion, rs9939609-A may be associated with poor glycemic control. These results also raise the possibility that other variants previously identified for T2D susceptibility could have a role in glycemic control. Future studies should assess the generalizability of these findings across diverse ancestries using independent cohorts and cross‑ancestry meta‑analyses. Furthermore, larger studies incorporating medication and treatment stratification would be necessary to test potential fine-grained pharmacogenomic hypotheses.

## Study limitations

Although this study has important strengths, including a well characterized cohort with key variables such as treatment regimens and years living with T2D available for modeling, it also has several limitations. The sample size limited our ability to perform stratified or interaction analyses that could clarify whether specific medications or combinations of therapies influence the association between rs9939609-A and glycemic control. We also did not include ancestry informative markers to quantify individual ancestry; thus, some residual confounding may remain. We also lacked detailed data on treatment adherence, disease severity, and diet, lifestyle or other factors that may influence the outcome. However, we attempted to partially account for these factors by adjusting for years living with T2D, although of course it is expected that some confounding persists. Finally, the moderate to small sample size may introduce imprecision into the effect estimates. Replication in a similar cohort is needed to assess the robustness of these findings.

## Data Availability

All aggregated data supporting the findings of this study, including allele and genotype frequencies and regression model outputs, are included within the manuscript. Individual‑level genetic data cannot be deposited in a public repository due to ethical restrictions. In accordance with Institutional Review Board approval, these data are available from the corresponding author upon reasonable request (nicolas.bargas@uib.no).
